# The interactions and hierarchical effects of long‐term agricultural stressors on soil bacterial communities

**DOI:** 10.1111/1758-2229.13106

**Published:** 2022-08-04

**Authors:** Shorok B. Mombrikotb, Maaike Van Agtmaal, Emma Johnstone, Michael J. Crawley, Hyun S. Gweon, Robert I. Griffiths, Thomas Bell

**Affiliations:** ^1^ Department of Life Sciences Imperial College London, Ascot Berkshire UK; ^2^ Louis Bolk Instituut Bunnik The Netherlands; ^3^ School of Life Sciences University of Warwick Coventry UK; ^4^ School of Biological Sciences University of Reading, Whiteknights Reading UK; ^5^ UK Centre of Ecology & Hydrology Bangor UK

## Abstract

Soils are subjected to multiple anthropogenic modifications, but the synergistic impacts of simultaneous environmental stressors on below‐ground communities are poorly understood. We used a large‐scale (1152 plots), long‐term (26 years), multi‐factorial grassland experiment to assess the impact of five common agricultural practises (pesticides, herbicide, liming, fertilizers and grazing exclusion) and their interactive effects on the composition and activity of soil microbial communities. We confirmed that pH strongly impacts belowground communities, but further demonstrate that pH strongly mediates the impacts of other management factors. Notably, there was a significant interaction between liming and the effect of pesticide application, with only half of the taxa responding to pesticide being shared in both limed and unlimed treatments. Likewise, nutrient amendments significantly altered bacterial community structure in acidic soils. Not only do these results highlight an hierarchy of effect of commonly used agricultural practices but also the widespread interactions between treatments: many taxa were significantly affected by interactions between treatments, even in the absence of significant main effects. Furthermore, the results demonstrated that chemical amendments may not percolate deeply into physically unperturbed soils with effects concentrated between 0 and 30 cm, despite 20+ years of treatment. The research shows that future changes to agricultural practices will need to consider interactions among multiple factors.

## INTRODUCTION

The role of microorganisms in terrestrial systems has been well described (e.g. Baldrian et al., 2012; Balser et al., 2002; Fitter et al., 2005; van der Heijden et al., 2008). Agricultural land accounts for approximately 38% of total land area (FAO, 2020) and is expected to further increase by 5% by 2050 (FAO, 2009); thus subjecting significant amounts of terrestrial systems to physical and chemical modification. To meet the demands of >9 billion people in 2050, land‐use intensification is expected to increase, including total fertilizer (+36% prediction of N + P_2_O_5_ + K_2_O; FAO, 2017) and pesticide usage (which increased 35% between 2001 and 2015; Maggi et al., 2019). The impact of land‐use intensification on soil microbial communities is poorly understood because of the complexity of the communities and of the soil environment, as well as the diversity of agricultural practices.

Previous work has shown that land use can significantly impact microbial communities (Bossio et al., 1998; Bossio et al., 2005; Rodrigues et al., 2013; Steenwerth et al., 2002; Thomson et al., 2015). These changes may be attributed to the substantial impact of agriculture on physical, chemical and biological properties of the soil (Jangid et al., 2008) rather than from land use itself (Lauber et al., 2008). Given that edaphic variables are strong drivers of community composition (Fierer et al., 2009; Fierer & Jackson, 2006; Griffiths et al., 2011) agriculture can significantly alter patterns of microbial composition and functioning. Most land management practices simultaneously alter multiple factors and although many studies have investigated the individual, and sometimes combined impact of some agricultural practices (e.g. liming and fertilizer application; Kennedy et al., 2005), only a few long‐term field studies have simultaneously tested the importance of biotic and abiotic factors and their interactive effects (Chu et al., 2007; Marschner, 2003; Sessitsch et al., 2001; Witter et al., 1993; Zhalnina et al., 2014; Zhang et al., 2005). There is a strong need to study long‐term and large‐scale field experiments that simultaneously manipulate multiple factors in order to provide a realistic understanding of their impacts and consequences.

This study addresses key management concerns for situations where multiple stressors are applied simultaneously. In these cases, there is the potential for interactions among the stressors, resulting in outcomes that could not be predicted by looking at the individual stressors in isolation (Matthaei et al., 2010; Preston, 2002; Townsend et al., 2008). Predicting the outcome of these interactions (synergistic/antagonistic) is particularly challenging in soil microbial communities when, for example stressors have different modes of action, preventing compensation by functionally redundant groups. Non‐additive effects of stressors that result in inconsistent impacts might be more difficult to manage because there must be a triage based on a valuation, for example liming increases plant yield but when pesticides are applied to protect those crops, there is a loss in beneficial rhizosphere microbes. In fact, there have been calls for better understanding of multiple stressors, particularly pesticides, using fully factorial experiments to understand interaction on soil microbial communities (Beaumelle et al., 2021). As well as addressing this, this study benefits from being one of few long‐term soil experiments.

Initiated in 1992, Nash's Field grassland experiment (Silwood Park, Berkshire, UK) has maintained five treatments across 1152 experimental plots: rabbit grazing prevention, pesticide application (insecticide, molluscicide, combination of insecticide and molluscicide), liming, herbicide and nutrient addition (12 combinations of N, P, K, Mg). We use this long‐term grassland experiment to study the interactions among multiple stressors focusing on the soil bacterial communities (SI 1). Some of these treatments, such as fertilizers and liming, are known to influence microbial communities [e.g. (Chu et al., 2007; Marschner, 2003; Pankhurst et al., 1996)]. Previous work on Nash's Field has demonstrated interactive effects of treatments on biogeochemical cycles (Macdonald et al., 2015), carbon utilization (Macdonald et al., 2015), carbon sequestration (Fornara et al., 2013) and the invertebrate community (Allan & Crawley, 2011). Here, we conducted the first survey of soil bacteria across the full experiment (1152 plots) to identify interacting effects of treatments and investigate how drivers of bacterial community composition persist after 20+ years of treatment. Additionally, as little is known regarding whether the effects of long‐term amendments penetrate beyond the soil surface and whether interactive effects are maintained, we assess how the effect of key treatments penetrate and persist vertically through the soil matrix. We hypothesise a hierarchical effect of treatments, where the magnitude depends on how the treatment directly impacts the microbial community. We predict that pH will act as a master regulatory due to its direct effect, while other treatments with indirect effects will disproportionally influence the community. A priori knowledge of the experimental site and treatments, whether they are thought to have direct or indirect impacts, leads us to predict a hierarchy beginning with pH (chemical/direct), ending with grazing (physical/indirect) and with pesticide (chemical/direct and indirect), nutrients (both direct and indirect) and herbicide (indirect) being intermediatory between the two.

## DISCUSSION

**FIGURE 1:**
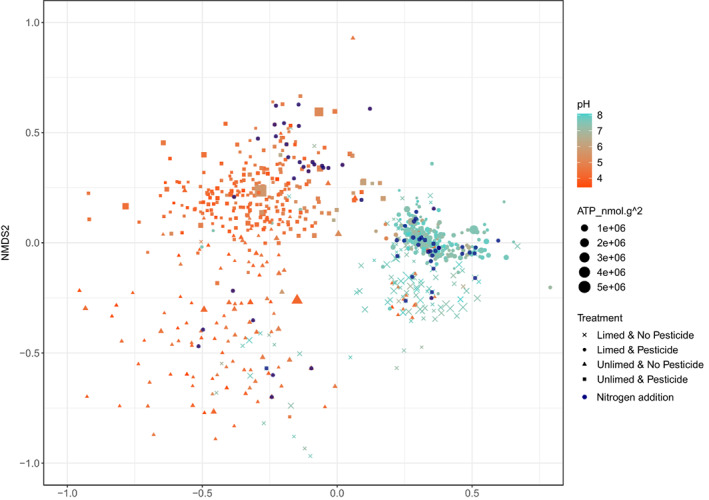
Non‐metric multidimensional scaling analysis (Bray–Curtis dissimilarity; stress = 0.14) of soil bacterial communities within Nash's field, illustrating the impact of liming and pesticide treatments on community structure. Unlimed treatments (filled triangles and filled squares) showed greater variance compared to limed treatments (filled circles and crosses); homogeneity of multivariate dispersions 0.28 and 0.18, respectively. Colour of the points represent pH (red/orange are acidic and blue/green are neutral) and size of the points represents the metabolic activity (ATP in nmol g^−1^; squared for illustration) of the soil microbial community. Pesticide application (insecticide or molluscicide) is illustrated by filled circles and filled squares and showed a decrease in variance (Table SI 2.2). Overlaid navy blue points represent soils with no nutrient additions for reference

Increased anthropogenic activity in terrestrial ecosystems will result in simultaneous impacts of multiple stressors. Results confirmed that pH strongly impacts belowground communities (ANOSIM *R*: 0.61, *p* < 0.001; Figure 1) but demonstrate multilevel interactions among stressors that differ substantially depending on pH. Overall, pesticide application had the strongest effects on the bacterial community structure out of the other experimental factors in both unlimed and limed soils (ANOSIM *R* = 0.58 and 0.48 respectively, *p* < 0.001; Figure 1). Specially, insecticides in neutral soils (ANOSIM *R*: 0.36, *p* < 0.001; SI 2) had a marginally greater effect than the molluscicide (ANOSIM *R*: 0.29, *p* < 0.001 SI 2) treatments, whilst in acidic soil the trends were reversed (Insecticide: ANOSIM *R*: 0.44, *p* < 0.001; Molluscicide: ANOSIM *R*: 0.47, *p* < 0.001; SI 2). Using a hierarchical ANOSIM with pH and pesticide treatments as the first and second levels (SI 2), the three remaining treatments showed varying degrees of influence on bacterial community structure (SI 2). Nutrient addition only influenced bacterial community structure in acidic soils, similar to previously published findings (Cassman et al., 2016), with a greater effect when no pesticides were added to the soil (SI 2). The impact of rabbit grazing on bacterial community structure was observed in both acidic and neutral soils but had a marginally greater effect in neutral soils (SI 2). Pulse herbicide treatment, which was applied at the beginning of the experiment but discontinued after 1994, had no overall effect at the top of the hierarchy, but its effect could still be observed in limed (neutral) soils when no pesticides were added (SI 2). These findings, therefore, suggest that the impacts of future anthropogenic change can only be interpreted in light of interactions among multiple stressors. Chemical amendments (liming, pesticides and herbicides) appeared to directly impact bacterial communities, while plant‐specific treatments (rabbit grazing) were less significant for determining bacterial community composition (SI 2). The response to inorganic fertilizers was pH‐dependent, which also modified the impact of pesticide on soil activity and bacterial community composition (SI 2). For example, we would not have observed any significant impact of nutrients on bacterial community structure if the experiment was conducted only at neutral pH (Figure 1: overlaid red points). The interactions that we observed among the treatments pose a challenge for the search for microbial indicators of soil health and demonstrate the need for experiments that simultaneously manipulate several factors.

Microcosm experiments provided evidence that the pesticides used here have a strong (and previously uncharacterised) direct impact on soil microbial communities that can be detected within short time frames (3 days; SI 3), and that is independent of any possible indirect effect via invertebrates; for example, by impacting belowground insects that in turn have an impact on soil properties. Treatment effects in the microcosms were qualitatively similar to those observed in the field experiment, with pH as the primary driver of community structure (ANOSIM: *R* = 0.745, *p* < 0.001; SI 3), followed by pesticide treatment (ANOSIM: *R* = 0.196, *p* = 0.019, SI 3). Overall, these results support the notion of a treatment‐dependent coupling between above‐ and below‐ground community composition (Fierer & Jackson, 2006), illustrating the hidden impacts of some agricultural treatments on soil communities that do not act through the plant component of the community.

Our results, together with previously published results using the same experiment, imply that pesticides have impacts that can percolate throughout the food web, indirectly altering plant communities by removing grazing pressures and directly altering bacterial community composition (Macdonald et al., 2015). Pesticides may also indirectly influence the belowground community through changes in soil characteristics following the loss of targeted pests. For example, greater organic matter turnover has been previously shown in these pesticide‐treated plots (Macdonald et al., 2015), perhaps because invertebrate grazing tends to increase litter inputs to the soil via plant damage and/or death (Olofsson et al., 2007) or because of changes to the plant communities (Allan & Crawley, 2011). Nonetheless, the treatments that had the strongest impacts on plant communities (grazing and nutrient additions) had only small impacts on belowground microbial communities. For example, rabbit grazing is a key regulator of grassland plant productivity and diversity in this system (Allan & Crawley, 2011), so their indirect impacts on belowground communities may be particularly prominent. However, rabbit grazing was only weakly linked to belowground activity and community structure compared to the other treatments. Based on these findings and previously published results, we suggest that alterations in edaphic characteristics are likely to have stronger effects on soil bacterial communities than plant manipulations (Girvan et al., 2003; Lauber et al., 2008).

Our experimental findings support the observational work demonstrating a strong role for pH on microbial communities (Fierer et al., 2009; Fierer & Jackson, 2006; Griffiths et al., 2011; Lauber et al., 2009) with liming resulting in community dissimilarity of *R* > 0.61 (Figure 1) and resulting in an approximately 1.5‐fold increase in metabolic activity (ATP, nmol g^−1^; *F*_1,6_ = 10.21, *p* = 0.019; SI 4). In addition to the strong direct impacts of pH, the results highlight how pH interacts with other biotic and abiotic factors. Although the carbon pool within the soil of Nash's Field was not found to be significantly different between pH treatments (Fornara et al., 2013), soil pH affects the mineralisation, concentration and bioavailability of substrates within the soil matrix (Kemmitt et al., 2006), therefore providing the means by which pH can modify the impact of some of the other chemical treatments (pesticides, nutrients). The molluscicide with no nutrient additions resulted in weak reductions in metabolic activity in acidic soils (decrease of 1.6%), but strong reductions in neutral soils (decrease of 56%). One possibility is that pH alters community composition resulting in taxa that are either more or less susceptible to the other treatments. Alternatively, changing the pH might directly interact with the treatments, for example making nutrients more bioavailable at different pH levels. We believe distinguishing between these alternatives will be an important component of future research.

Nutrient additions altered the metabolic functioning of communities (*F*_11,396_ = 2.5, *p* < 0.001; SI 4), but the impact of the nutrients also differed between pH treatments [*F*_11,396_ = 3.7, *p* < 0.001; Figure 1(B); SI 4]. While P, K and Mg independently reduced community metabolic activity in neutral soils, all other nutrient combinations increased activity. We suggest this increased activity in N‐treated neutral soils is mediated by the impact of N on plant belowground biomass and root‐associated activity (Zhu et al., 2016). Conversely, in acidic soils, the addition of N alone and the combination of N, K and Mg resulted in a decrease in metabolic activity while all other nutrients increased activity relative to controls (SI 4). The result contrasts with previous work that has suggested that N additions can facilitate fast growers which utilize labile deposits, thereby leaving soil organic carbon and increasing carbon stocks (Liu & Greaver, 2010). In support of this hypothesis, Fornara et al. (2013) showed nitrogen addition to soils increased the carbon stock and nitrogen retention. Next‐generation sequencing data (on a subset of plots) demonstrated that 25% of OTUs assigned to Acidobacteria were significantly affected by liming. Acidobacteria, typically slow growers (Kielak et al., 2016), are further enriched in N‐treated soils, with long‐term fertilizer usage increasing their abundance (Ma et al., 2018). Post hoc checks revealed this could be because the N application further acidified the soil (*x̄* decrease in pH of 0.4 ± SE 0.08 and 0.07 ± SE 0.04 in unlimed and limed soils, respectively), which would favour Acidobacteria. Thus, while increased metabolic activity might occur over short time scales, continued application of N leading to soil acidification will select for slow‐growing communities and a reduction in metabolic activity (Wei et al., 2013). This indirect impact of N inputs in acid soils could also lead to build‐up of organic matter if growth is unable to keep up with new inputs. Our results clearly show that different types of soil communities (which we now know are strongly related to soil pH and associated factors) respond differently to various types of agricultural amendments. Predicting the global ecological impacts of agricultural improvements, therefore, needs to take into account parent material, liming management and climate‐driven pH‐related characteristics on the native microbial communities.

**FIGURE 2:**
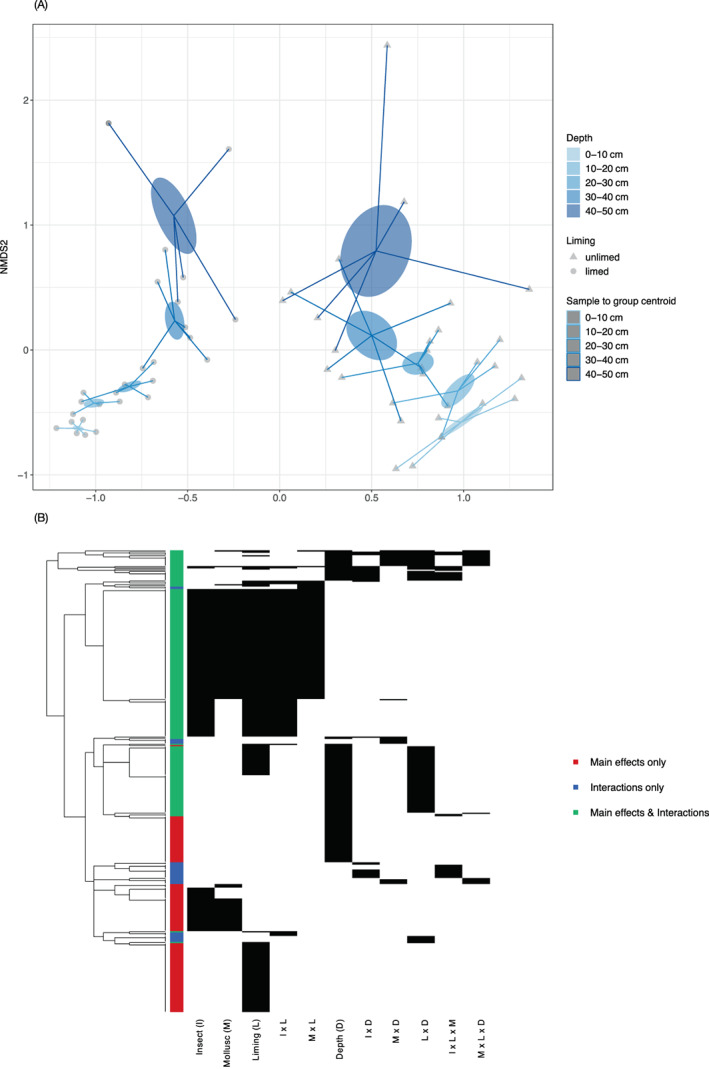
Dissipation of treatment impacts on soil composition and impact of treatment on individual taxa (class). Changes in bacterial community profiles in different pH and pesticide (insecticide and molluscicide) treated soils within Nash's field with depth (0–50 at 10 cm intervals). (A) Non‐metric multidimensional scaling analysis (Bray–Curtis dissimilarity; stress =0.08) distinguishing between limed (circles) and unlimed (triangles) with colour darkening with depth and (B) Heatmap of significant treatments on individual taxa, across all depths, distinguishing the main effect only (red), interactions only (blue) and both main and interaction effects (green)

To enable analyses of the entire Nash's field experiment, including all replicates and treatments, we used a simple TRFLP approach; however, more detailed analyses of taxonomic responses were focused on the liming and pesticide treatments (which had the greatest effect according to the TRFLP) using amplicon sequencing. Additionally, this allowed us to test for the wider effects of these treatment effects with depth. The sequencing confirmed the hierarchical effects of treatments observed in the TRFLP and additionally demonstrated that though effect size reduces with depth, the impacts of these management practices are maintained in the top 30 cm. Results showed a decrease in bacterial community dissimilarity with depth such that only a marginal difference between the two liming treatments was observed at 40–50 cm [Figure 2(A)]. This is likely due to chemical impacts from aboveground applied amendments such as liming and pesticides decreasing with depth (Gonçalves & Alpendurada, 2005). Particular taxa were significantly influenced by depth [Figure 2(B)] and there were significant interactions between depth, pesticide and liming. Notably, *Chlorobi (OPB56)*, an obligately anaerobic photoautotrophic bacterium, showed opposing trends with depth depending on the pesticide treatment; in control and molluscicide soils, it decreased with depth, while in the insecticide plot it increased with depth. Similarly, *Firmicutes*, which can confer disease protection for some plants, decreased with depth in control plots but generally increased with depth in molluscicide and insecticide‐treated soils. This is contrary to other studies that found increased in *Firmicutes* with Dazomet (fumigant used as an herbicide/fungicide; Feld et al., 2015). Our findings show that key groups of bacteria that are often used as soil health indicators because of their positive impact on plants and disease suppressiveness (Berendsen et al., 2012), such as *Firmicutes*, could be sequestered to deeper soils with commonly used management practices; however, this may be pesticide‐specific. As studies have shown certain groups of microbes are able to utilize the pesticide as an energy source (Chun‐lei et al. 2013), in some cases breaking it down to residual concentration (Thomas et al., 2017), whilst being toxic to other groups (Johnsen et al. 2001), gauging the impact of pesticides on microbial communities remains challenging. Future studies should quantify the concentration of pesticides and their bi‐products to gauge their retention within the soil matrix.

Clustering of the sequencing reads resulted in 8508 OTUs (97% cutoff) which were assigned to 111 classes. We conducted nested analysis of variance using the relative abundance of each OTU as a response variable, and depth, pesticides (levels: control, insecticide, molluscicide), liming (2 levels: limed, unlimed), and their interactions, as predictor variables. We found that 382 OTUs were significantly affected by one or more of the treatments [Figure 2(B)]. Of the 382 OTUs that were significantly impacted, 135 were impacted by the main effects of the treatments only, while the remainder were impacted by interactions among treatments only (32 OTUs) or by both main effects and interactions (215 OTUs) [Figure 2(B)]. Interactions among treatments, therefore, played a prominent role in the response of the majority (65%) of OTUs impacted by the treatments. Similar results have been found in other multiple stressor studies (e.g. Romero et al., 2020), leading to similar conclusions that multiple‐stressor results in unpredicted shifts in the community composition from individual stressors. This is particularly pertinent when considering taxonomic indicators of external stressors, as these stressors are likely to invoke different taxonomic indicators across different soil contexts; results presented here show only 45% of taxa responding to pesticides (both insecticide and molluscicide) were shared across limed and unlimed treatments (SI 5) with some taxa being pesticide‐specific (SI 5). Given these results, using individual taxa as environmental stressors marker without soil‐specific knowledge will be challenging and a better understanding of interactive effect to model responses is needed.

Grasslands and agroecosystems contain complex networks of interactions occurring at multiple levels, across multiple organisms and encompassing both biotic and abiotic variables. Our results highlight widespread interactions between treatments on the activity and composition of soil microbial communities, which substantially complicates the use of indicator species for soil health assessments. These interactions were particularly strong for the liming and pesticide treatments and indicated the likely simultaneous direct and indirect treatments of communities. These effects will likely be observed across different systems with additional interactions with other agricultural practices, for example, soil rotation will aid deeper penetration of chemical stressors increasing effect size and duration. The research shows that future changes to agricultural practices will need to take into account interactions among multiple factors and account for differential responses. Overall, the results suggest that soil pH might be viewed as a master regulator of soil microbial communities by mediating the impacts of other factors, including pesticide application, rabbit grazing and nutrient addition. Characterizing the generality of these interactions will be an important component of predicting how belowground communities will respond to changing land‐use practices.

## AUTHOR CONTRIBUTIONS

S.B.M., T.B. and R.I.G. conceived the ideas, designed the sampling methodology, analysed the data and led the writing of the manuscript. S.B.M. conducted the experiments, collected and processed all the samples. E.J. assisted in sample collection of the complete survey. M.V.A. conducted the vertical profile study alongside S.B.M. M.J.C. designed the field experiment. H.S.G. conducted the bioinformatics for the vertical profile survey. All authors contributed to drafts and gave final approval for publication.

## Supporting information


**APPENDIX S1** Supporting Information.

## Data Availability

Raw data will be publicly accessible through Data Dryad (http://datadryad.org/).
