# Supplementary material for: The interactions and hierarchical effects of long‐term agricultural stressors on soil bacterial communities
Source: Environ Microbiol Rep. 2022 Aug 4;14(5):711–8. doi: 10.1111/1758-2229.13106 (PMC9804416; doi:10.1111/1758-2229.13106)
Supplement: Supplementary file 1 — APPENDIX S1 Supporting Information. [file EMI4-14-711-s001.docx]

**Supplementary Information**

**SI 1: Methods and materials**

**SI 2: Hierarchical ANOSIM and community variance**

**SI 3: Direct impact of pesticides on soil composition and activity in a microcosm experiment.**

**SI 4: Metabolic activity (ATP nmol g^-1^) of soil microbial communities within Nash’s Field.**

**SI 5: Pesticide indicator taxa across limed and unlimed treatments in Nash’s Field.**

**SI 1: Method and materials**

***Study site and experimental design***

Nash’s Field (Silwood Park, Berkshire, National Grid reference 41/944691) is a long-term grassland experiment initiated in 1992. The experiment consists of 1152 experimental plots (2 x 2 m) where soil pH, grazing, pesticides, herbicides, and nutrients have been manipulated. Details of the experiment design are given below. The site, measuring approximately 6 ha, has acidic sandy soil with vegetation dominated by *Agrostis capillaris*, *Anthoxanthum oduratum*, *Festuca rubra* and *Holcus mollis* (Edwards *et al*. 1999).Vegetation is grazed extensively by rabbits, and molehill disturbance has also been observed at the site (Edwards *et al*. 1999). Nash’s Field is surrounded by woodlands (Oak, *Quercus robur* and Birch, *Betula pendula*) and bracken.

The experiment design is described in detail in Edwards *et al*. (1999). Briefly, the experiment consists of a randomised block split-plot design (Fig SI 1.1). Whole blocks annually receive insecticide (240 g ha^-1^ chlorpyrifos and 336 g ha^-1^ dimethoate), or molluscicide (slug pellets containing metaldehyde; 960 g ha^-1^ active ingredients), or a combination of insecticide and molluscicide (hereafter referred to as “both” treatment), or neither (hereafter "control") and are replicated twice (n = 8 blocks; K, L, M, N, P, Q, R and S). Within each block, half is fenced to prevent rabbit grazing (3 cm mesh wire fence, 1 m aboveground), and half remains unfenced. Within each of the fenced/unfenced plots, half is treated with lime (CaCO_3_; 20 t ha^-1^) to increase pH from 4.56± 0.02 (mean ± standard error throughout) to 7.53±0.02. Within each pH treatment, one-third was treated with an herbicide to remove grasses (sethoxydim at 870 g active ingredient ha^-1^), one third to remove herbs (dicamba + MCPA + mecoprop at 260, 2080, 4160 g active ingredient ha^-1^ respectively), and one third was left untreated. The herbicide treatment was started in 1992 and suspended in 1994. Within each herbicide treatment, there are 12 plots (2 x 2 m) each receiving one of 12 nutrient combinations of N ammonium nitrate (150 kg ha^-1^), P as Triple Superphosphate (35 kg ha^-1^), K as potassium muriate (225 kg ha^-1^) and Mg as magnesium sulphate (11 kg ha^-1^). In total, the design gives 1152 individual 2 x 2 m plots with two independent replicates of each treatment level combination.

**Fig SI 1.1:** Schematic of the experimental design of Nash’s Field, outlining the nested design (Allan & Crawley 2011).

**
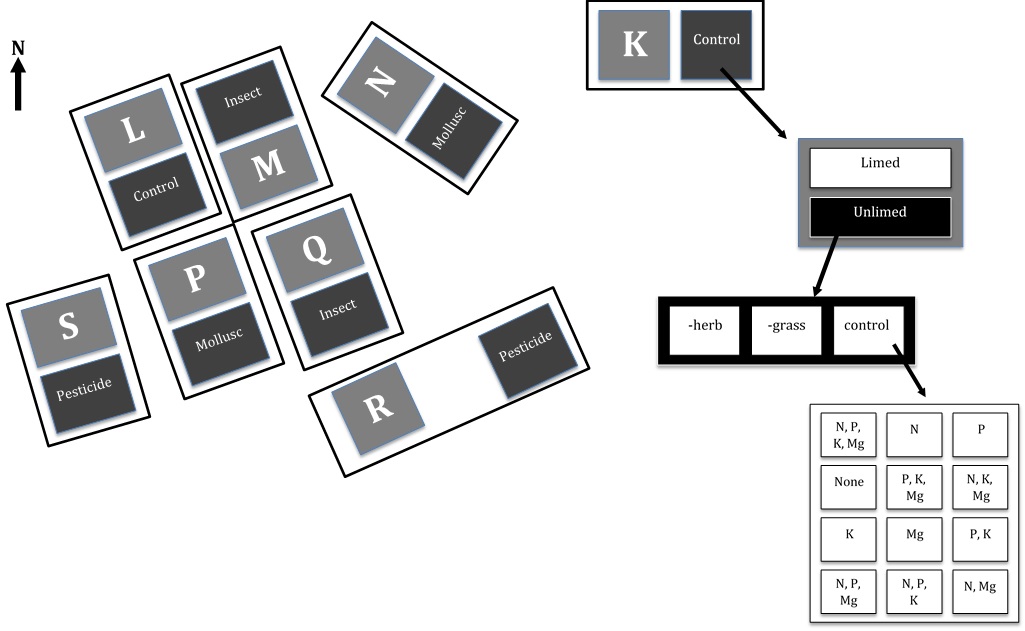
**

***Complete soil bacterial survey***

Because of the large number of plots in the experiment, we first used DNA fingerprint techniques to identify the treatments that had the strongest impacts on community structure across all 1152 plots. Soil samples were collected using a sterile polyethylene corer (1.5 x 10 cm) from the top 10 cm of all plots within Nash’s Field between August and September 2013. Four cores were collected from each plot approximately 1 m apart from the centre of the 2 x 2 m plot, mixed and homogenised by hand. All soil samples were stored in micro-centrifuge tubes at -80°C on the day of collection. Soil moisture was calculated as the percent water content, based on mass lost between wet (on day of collection) and dry mass (3 days at 70°C). Soil pH for all plots was measured in 2007, but has been periodically checked to ensure pH is maintained, with no significant difference due to time found between 2007 and a 2017 subset (paired t-test; t=-0.045, df=15, p=0.96).

Total nucleic acids were extracted from soils (wet weight 0.1-0.94 g; $\bar{x}$ 0.371 ± SE 0.0046 g) using Zymo ZR-96 Soil Microbe DNA Kit (Zymo Research, Irvine, CA, USA) following manufacturer’s guidelines. Bacterial community profiles were assessed using TRFLP analysis as described in Griffiths *et al*. (2011). Briefly, bacterial 16S rRNA genes were amplified (V1-V3 variable region; 63F and 519R primers) with 6FAM fluorescent dye tagged forward primer (5’ end; Griffiths *et al*. 2011). Amplicons were purified using Zymo Clean-Up Kit (Zymo Research, Irvine, CA, USA) following manufacturer’s guidelines then digested using restriction endonuclease *Msp1* (3000 units). After amplicon purification, samples were digested using restriction enzyme *Taq1* (3000 units). Gel capillary fragment analysis on digested product was conducted alongside Liz600 size standard. Samples were analysed with GeneMarker (SoftGenetics, PA, USA), manually binned (between 50bp and 550bp) and visually quality checked (threshold of intensity 40 applied). Any samples that were too noisy to detect peaks were removed. Intensity of fragments were converted to relative proportion per sample.

***Soil metabolic activity***

We measured the potential metabolic activity in each sample by quantifying the amount of ATP in the samples. On the day of collection, soil samples were washed to extract microbial cells. Sterile phosphate buffer saline (PBS; Sigma P4417; pH 7.4, at 25 °C) was added to soils in equal parts (soil weight: PBS volume), vortexed (~10 seconds) to saturate the soils, then shaken on a table top shaker for 10 minutes at 250 rpm. The supernatant was used to measure the overall metabolic activity of the community using the BacTiter-Glo^TM^ Microbial Cell Viability Assay (Promega: G8230) to quantify adenosine triphosphate (ATP) of each sample. The reagent contains the thermostable luciferase enzyme that binds to ATP molecules in the presence of oxygen and magnesium, emitting a luminescent signal that can be detected using a plate reader (BioTek Synergy2; Serial: 266207). Reagent (2:1 ratio of BacTiter-Glo^TM^ reagent to supernatant of the soil washes after a 50-fold dilution in PBS) was dispensed into each well at 250 μL sec-1, followed by a 5 second medium shake. Samples were kinetically monitored for 7 minutes, with luminescent readings taken every 1.15 minutes and exposure time set to 0.5 seconds. The maximum luminescence detected per sample was standardised against the control by subtracting the mean luminescence of the control from each sample. We found that pH had an effect on the assay. We therefore corrected for the pH effect by conducting calibration curves with known concentrations of ATP. Luminescence was converted to nmol g^-1^ of ATP for both pH 5 and pH 7 using the following calibration:

*Equation 1*

$$\mathrm{ATP}\left( \mathrm{nmol}g^{-1} \right)= \left( \frac{\mathrm{luminescence}_{sample}-\mathrm{luminescence}_{control}}{x} \right) x dilution factor$$

Where $x$ is 1416.8 for pH 5 soils and 1693.1 for pH 7 soils (SI 2).

***Targeted amplicon sequencing***

We used 16S amplicon sequencing on the MiSeq platform to obtain more detailed assessments of communities from the treatments that had been identified as having strong effects. Soil samples were collected using a metal 60cm corer (1.2cm diameter) from the centre of 16 plots within Nash’s Field in October 2015; all pesticide treatments and both limed and unlimed treatments within unfenced, no herbicide and no nutrient blocks. These plots were selected by focusing on treatments that had the largest effect according to the TRFLP (pesticide and liming) and using all and no nutrients to provide extreme of the nutrient addition treatment and provide replicate for the nested design. We specifically examine the effect of depth, the core was separated into 10cm section from the top down unto 50cm and each section was treated independently. As with the complete survey, soil moisture was measures as percentage water content and soil frozen at -80°C on the day of collection.

Total nucleic acids were extracted from soils (wet weight 0.07-0.65 g; $\bar{x}$ 0.33 ± SE 0.015 g) using Zymo ZR-96 Soil Microbe DNA Kit (Zymo Research, Irvine, CA, USA) following manufacturer’s guidelines. The V4 region (~250 bp) of the bacterial 16S rRNA genes were amplified using the dual indexing PCR protocol described in Kozich *et al*. (2013), allowing 364 samples to be uniquely indexed. Briefly, 1 μL of nucleic acid was used as the template in a 50 μL reaction volume consisting of 0.5 μL Q5 High Fidelity Taq (2000 unit ml^-1^), 10 μL 10X reaction buffer, 10 μL GC enhancer, 1 μL 10 mM dNTP, 22.5 μL molecular grade water and 5 μL of dual indexed primer (0.125 μM of each forward and reverse primer). Parameters for the PCR were as follows; initial denaturing at 95°C for 2 minutes followed by 25 cycles of 15s at 95°C, 15s at 55°C and 30s at 72°C, with a final extension time of 10 minutes at 72°C. Amplifications were confirmed on 1% agrose gel stained with GelRed™ (Biotinium, Inc. Fremont, CA, USA). Amplicons were normalized to up to 25 ng per sample using SequalPrep Normalisation Plate Kit (Thermo Fisher Scientific, Loughborough, UK) and libraries were pooled per plate (96 samples). The 4 libraries were quantified using Qubit High Sensitivity (Thermo Fisher Scientific) and pooled in equal concentration. 400 pM library and 40 pM PhiX (Illumina, Inc., San Diego, CA, USA) control were prepared and denatured with 2 μL 2 N NaOH (Sigma-Aldrich, Gillingham, UK) for 5 minutes at room temperature then neutralized with 2 μL 2 N HCl (Sigma-Aldrich). 8 pM library with 10% PhiX control was created with chilled HT1 buffer and loaded into the V3 chemistry MiSeq cartridge (Illumina, Inc.) to achieve 2x300bp sequencing reads.

Sequenced paired-end reads were joined using PEAR (Zhang *et al*. 2014), quality filtered using FASTX tools (Hannon, <http://hannonlab.cshl.edu>), presence of PhiX and adapters were checked and removed with BBTools (jgi.doe.gov/data-and-tools/bbtools/), and chimeras were identified and removed with VSEARCH_UCHIME_REF (Rognes *et al*., 2016) using Greengenes Release 13_5 (97%) (DeSantis *et al*. 2006). Singletons were removed and the resulting sequences were clustered into operational taxonomic units (OTUs) with VSEARCH_CLUSTER_FAST (Rognes *et al*., 2016) at 97% sequence identity (Tindall *et al*. 2010). Representative sequences for each OTU were taxonomically assigned by RDP Classifier with the bootstrap threshold of 0.8 or greater (Wang *et al*. 2007) using the Greengenes Release 13_5 (full) (DeSantis *et al*. 2006) as the reference. Unless stated otherwise, default parameters were used for the steps listed. Sequences were subsequently rarefied to 5000 OTUs and OTUs with fewer than 2 reads were removed.

***Direct impact of pesticides on microbial communities in microcosms***

We conducted microcosm experiments in the lab to assess whether the pesticides used in the field experiment had direct effects on the soil microbial communities. Bulk soil collected from the top 20 cm of soil (GPS: 51.41327N, 000.643821W), adjacent to Nash’s Field, was sieved partially dry (<2mm sieve), autoclaved (121°C for 15 minutes) twice to sterilise at least 24 hours apart and dried for 24 hours at 70°C. Sterile soil was soaked in sterile phosphate buffer solution (pH 5 or 7) in equal ratio (ml:g) and shaken vigorously to ensure the soil was fully saturated by the buffer. Soil/buffer mix was left overnight before draining and dried at 70°C, followed by a final autoclave. Sterile pH manipulated soil (pH 5 and 7) was further treated with pesticides; metaldehyde, chlorpyrifos, and dimethoate. Pesticides were added at approximate quantities as Nash’s Field study site. Molluscicide slug pellets containing the active ingredient metaldehyde (TRIGGER 3 MAPP 14304; 3% w/w metaldehyde) were ground in a bead beater (20 Hz for 5 minutes). 0.36 g of powered slug pellets were dissolved in 1 L of sterile water. Insecticide consists of two active ingredients; dimethoate (Danadim Progress MAPP 15890; 400 g/L) and chlorpyrifos (Pontoon 48EC MAPP 14555; 480 g/L). 356.25 μL of each dimethoate and chlorpyrifos was diluted in 1 L of sterile water (171 μg ml-1 and 142 μg ml-1 active ingredient respectively). Soils were soaked in sterile pesticide solution (1:1 ratio solution to weight) for 48 hours and shaken every 12 hours to saturate. After 48 hours soaking, soils were dried in sterile breathable autoclave bags at 70°C for between 24-48 hours then homogenised by hand. Along with an no pesticide control, the design gave a total of 6 treatments (two pH levels x 3 pesticide treatments) with 6 replicates for each treatment combination. Sterile treated soils were loaded into 1.2 ml deep well plates using a column loader (x̄ 0.45 ±0.01g per well).

Soil microbial community was washed (1:1, wet weight(g): PBS(ml)) from a single soil core (6 x 15 cm; bulb corer) from adjacent to Nash’s Field study site (National Grid Reference: SU9436569076) and stored in 20% glycerol at -80°C. 50 μL of soil wash community was inoculated into each soil filled microcosm, covered in breathable seals and incubated at 22°C for 3 days. After incubation, soils were washed by adding 450 μL of sterile PBS per well (1:1 wet weight(g): PBS(ml) based on the mean soil weight per microcosm x̄ 0.45 ±0.01g). Metabolic activity of the post incubation washed community was measured. As with the complete Nash’s field microbial survey, bacterial communities were profiled using TRFLP, as described above.

***Statistical analysis***

All data was analysed using R.Studio (Mac version: 0.98.501). Metabolic activity was analysed using a nested ANOVA design as described by Allan & Crawley (2011). Similarly, each classified OTU within the vertical profile dataset was analysed using a nested ANOVA. There are no equivalent multivariate techniques for analysing the impact of the treatments on microbial community structure with interaction and accounting for the split plot design. Although PERMANOVAs are typically used and can handle one treatment split plot; Nash’s Field consists of 5 treatment nested within each other, thus PERMANOVAs are unsuitable here. Instead, community structures were analysed using a series of nested ANOSIM tests. The ANOSIM R statistic quantifies the amount of variation in dissimilarity within treatments versus among treatments. Values of R close to zero indicate no evidence of a treatment effect on community composition, while values close to 1 indicate a strong treatment effect. ANOSIM tests were first conducted on each treatment individually. The treatment with the most significant ANOSIM p-value was selected, and we conducted ANOSIM tests of the remaining treatments within each level of the top-ranked treatment. This process was repeated, creating a nested hierarchy (SI 1.2). Where treatments had multiple levels (e.g. nutrient addition), the analysis was conducted in a binary fashion so that levels were grouped into *with treatment* or control. Multivariate data were visualised using non-metric multidimensional scaling (NMDS; Vegan packages). Multivariate homogeneity of dispersions was calculated using *betadisper* in Vegan. Multiple testing on analysis of variance of each taxa was corrected using Benjamini, Hochberg, and Yekutieli correction with the number of comparisons equal to the number of taxa.

**Fig SI 1.2:** Schematic representation of hierarchical ANOSIM.

The impact of molluscicide and insecticide appeared to be alleviated when applied simultaneously (Fig SI 1.3). The aboveground community also experienced little response to simultaneous application of insecticide and molluscicide (Allan & Crawley 2011). The two blocks (blocks R and S) with both molluscicide and insecticide additions are highlighted in the SI 1.2. The unfenced portion of Block R and S have become dominated by nettles and bracken, a plant community response that only appeared two decades after the experiment began. Because of the vast difference in plant community and, this block was eliminated from subsequent analysis.

**Fig SI 1.3:** Non-metric multi-dimensional scaling analysis of soil bacterial communities within Nash's Field in plot which received both molluscicide and insecticide treatments.

**SI 2: Hierarchical ANOSIM**

Hierarchical analysis of similarity (ANOSIM) using Bray-Curtis dissimilarity index for bacterial communities within Nash’s Field. Each of the five treatments applied within Nash’s Field are tested at each level of the hierarchy, with the hierarchy being determined by the overall effect size of each treatment at the top of the hierarchy. Fig SI 2.1 illustrates the results with the size of the circle represents the ANOSIM R statistic (only significant terms plotted).

**Fig SI 2.1:** Hierarchical analysis of similarity of soil microbial communities in Nash’s Field.

 Multivariate homogeneity of dispersions of soil bacterial communities within Nash's Field, quantifying the variation of the samples from the centroid of the group. Dispersion of each pesticide treatment within each liming treatment was tested and the difference to the respective control was calculated. Results are presented in Table SI 2.2.

**Table SI 2.2:** Sample variance (multivariate homogeneity of dispersions) of soil microbial communities in Nash’s field treated with liming and pesticides for >20 years.

|  | **Average distance to median** | | | |
| --- | --- | --- | --- | --- |
| **Liming** | **Control** | **Insecticide** | **Molluscicide** | **% Difference** |
| Limed | 0.23 | 0.13 |  | 43.97 |
|  |  |  | 0.15 | 35.02 |
| Unlimed | 0.29 | 0.24 |  | 20.15 |
|  |  |  | 0.22 | 23.66 |

**SI 3: Direct impact of pesticides on soil composition and activity in a microcosm experiment.**

To assess the direct impact of chemical amendments on soil bacterial communities, a previous unperturbed acidic soil community is inoculated into sterile pH and pesticide manipulated soil (22°C for 3 days) in a microcosm experiment. The treatments are equivalent to those used in Nash’s field study site and the experiment is used as a control comparison to ascertain whether similar trends can be seen when additional variables (e.g. plants) are eliminated. Result illustrate that chemical amendments have a direct impact on soil microbial communities and these can be seen in short timeframes. Fig SI 3.1 illustrates the results of pH and pesticides on A) community composition and B) metabolic activity.

**Fig SI 3.1** A) Non-metric multi-dimensional scaling analysis (Bray-Curtis dissimilarity) where the background (black points) is the residual DNA in sterile soil with no community inoculum (95% CI ellipse dashed line). Colours denote the pesticide treatment, and shapes denote the liming treatments (circles = pH 7, triangles = pH5. B) The metabolic activity (x̄ ATP in nmol g^-1^ ± SE) of the communities illustrating the direct impact of pesticide on the metabolic activity and the interaction with pH.

**SI 4: Metabolic activity (ATP nmol g^-1^) of soil microbial communities within Nash’s Field.**

Metabolic activity (ATP, nmol g^-1^) provided a snapshot of the metabolic potential and activity of communities and a measure of overall functioning to a particular condition. The assay allows the estimate of overall and general function of cells within the community. A nested ANOVA is used to analysed the results of the survey accounting for the split-plot design (Table SI 4.1). Results show the significant impact of liming and molluscicide, liming and nutrient addition on metabolic activity (Table SI 4.2).

**Table SI 4.1** Result of the Nested analysis of variance (ANOVA) of the metabolic activity (ATP nmol g^-1^) of microbial communities within Nash’s Field. Error refers to the which level in the split-plot the analysis is assessing.

| Error(Plot) |  |  |  |
| --- | --- | --- | --- |
|  | df | F | P |
| Insecticide | 1 | 1.51 | 0.31 |
| Molluscicide | 1 | 2.69 | 0.20 |
| Residuals | 3 |  |  |
|  |  |  |  |
| Error(Block) | |  |  |
|  | df | F | P |
| Block | 1 | 0.12 | 0.76 |
| Insecticide:Block | 1 | 0.07 | 0.81 |
| Molluscicide:Block | 1 | 0.96 | 0.40 |
| Residuals | 3 |  |  |
|  |  |  |  |
| Error(Plot:Block:Liming) | |  |  |
|  | df | F | P |
| Liming | 1 | 10.21 | 0.02 |
| Insecticide:Liming | 1 | 0.30 | 0.60 |
| Molluscicide:Liming | 1 | 6.37 | 0.05 |
| Block:Liming | 1 | 0.00 | 0.98 |
| Insecticide:Block:Liming | 1 | 0.00 | 0.98 |
| Molluscicide:Block:Liming | 1 | 0.01 | 0.94 |
| Residuals | 6 |  |  |
|  |  |  |  |
| Error(Plot:Block:Liming:Plants) | | |  |
|  | df | F | P |
| Plants | 2 | 0.18 | 0.84 |
| Insecticide:Plants | 2 | 1.87 | 0.18 |
| Molluscicide:Plants | 2 | 0.42 | 0.66 |
| Block:Plants | 2 | 2.17 | 0.14 |
| Liming:Plants | 2 | 1.74 | 0.20 |
| Insecticide:Block:Plants | 2 | 0.11 | 0.90 |
| Molluscicide:Block:Plants | 2 | 1.48 | 0.25 |
| Insecticide:Liming:Plants | 2 | 1.70 | 0.20 |
| Molluscicide:Liming:Plants | 2 | 1.95 | 0.16 |
| Block:Liming:Plants | 2 | 0.29 | 0.75 |
| Insecticide:Block:Liming:Plants | 2 | 0.95 | 0.40 |
| Molluscicide:Block:Liming:Plants | 2 | 0.44 | 0.65 |
| Residuals | 24 |  |  |
|  |  |  |  |
| Error(Plot:Block:Liming:Plants:Treatment) | | | |
|  | df | F | P |
| Treatment | 11 | 2.54 | 0.00 |
| Insecticide:Treatment | 11 | 1.67 | 0.08 |
| Molluscicide:Treatment | 11 | 0.94 | 0.51 |
| Block:Treatment | 11 | 1.36 | 0.19 |
| Liming:Treatment | 11 | 3.71 | 0.00 |
| Plants:Treatment | 22 | 1.29 | 0.17 |
| Insecticide:Block:Treatment | 11 | 1.06 | 0.39 |
| Molluscicide:Block:Treatment | 11 | 0.75 | 0.69 |
| Insecticide:Liming:Treatment | 11 | 2.22 | 0.01 |
| Molluscicide:Liming:Treatment | 11 | 0.90 | 0.54 |
| Block:Liming:Treatment | 11 | 0.44 | 0.94 |
| Insecticide:Plants:Treatment | 22 | 0.98 | 0.49 |
| Molluscicide:Plants:Treatment | 22 | 1.01 | 0.45 |
| Block:Plants:Treatment | 22 | 0.95 | 0.52 |
| Liming:Plants:Treatment | 22 | 1.02 | 0.44 |
| Insecticide:Block:Liming:Treatment | 11 | 0.53 | 0.88 |
| Molluscicide:Block:Liming:Treatment | 11 | 0.91 | 0.53 |
| Insecticide:Block:Plants:Treatment | 22 | 1.21 | 0.24 |
| Molluscicide:Block:Plants:Treatment | 22 | 0.59 | 0.93 |
| Insecticide:Liming:Plants:Treatment | 22 | 1.01 | 0.46 |
| Molluscicide:Liming:Plants:Treatment | 22 | 0.89 | 0.61 |
| Block:Liming:Plants:Treatment | 22 | 1.12 | 0.33 |
| Insecticide:Block:Liming:Plants:Treatment | 22 | 1.73 | 0.02 |
| Molluscicide:Block:Liming:Plants:Treatment | 22 | 0.67 | 0.87 |
| Residuals | 396 |  |  |

**Fig SI 4.2** The metabolic activity ($\bar{x}$ nmol g^-1^ ± SE) of the microbial community in communities treated with (left) pesticides treatment (insecticide or molluscicide) and (right) nutrients (N, P, K, Mg and 12 combination) and their interaction with liming.

**SI 5: Pesticide indicator taxa across limed and unlimed treatments in Nash’s Field.**

As described in SI 1, each classified OTU within the vertical profile dataset was analysed using a nested ANOVA with a Benjamini, Hochberg, and Yekutieli correction for multiple testing. This presented 382 OTUs with one or more significant treatments. These data was used to assess how many taxa that responded to pesticide were shared between limed and unlimed treatments. For each OTU that had a significant main effect of insecticide or molluscicide, the mean relative abundance with the top 10cm was used to determine whether taxa OTU responded, positively or negatively in both limed and unlimed. Using the taxonomic assignment of these OTUs, how many were unique to each pesticide treatment is also detailed.

|  | **Insecticide Indicator** | **Mollusc Indicator** |
| --- | --- | --- |
| **Number of OTUs in main effect** | 160 | 124 |
| **Number of OTUs responding in both pH treatments** | 56 | 72 |
| **Percentage of OTUs responding in both pH treatments** | 35% | 58% |
| **Number of taxa unique to Pesticide treatment (36 across both pesticide treatments)** | 7 | 9 |
| **Percentage of taxa unique to pesticide treatment responding in both pH treatments** | 19% | 25% |
|  |  |  |
| Taxa ID |  |  |
| Euryarchaeota Methanobacteria |  | Yes |
| Actinobacteria Thermoleophilia |  | Yes |
| Armatimonadetes Fimbriimonadia |  | Yes |
| Chloroflexi Ktedonobacteria |  | Yes |
| Chloroflexi S085 |  | Yes |
| FCPU426 unclassified |  | Yes |
| Planctomycetes OM190 |  | Yes |
| Planctomycetes unclassified |  | Yes |
| Tenericutes Mollicutes |  | Yes |
| Acidobacteria Solibacteres | Yes |  |
| Planctomycetes Phycisphaerae | Yes |  |
| Planctomycetes Pla3 | Yes |  |
| Planctomycetes vadinHA49 | Yes |  |
| Proteobacteria TA18 | Yes |  |
| TM7 TM7-1 | Yes |  |
| Verrucomicrobia Verruco-5 | Yes |  |
| Acidobacteria Acidobacteria-6 | Yes | Yes |
| Acidobacteria RB25 | Yes | Yes |
| Actinobacteria Actinobacteria | Yes | Yes |
| Bacteroidetes Saprospirae | Yes | Yes |
| Bacteroidetes Cytophagia | Yes | Yes |
| Chlamydiae Chlamydiia | Yes | Yes |
| Chlorobi SJA-28 | Yes | Yes |
| Chloroflexi Anaerolineae | Yes | Yes |
| Cyanobacteria 4C0d-2 | Yes | Yes |
| Elusimicrobia Elusimicrobia | Yes | Yes |
| OD1 ABY1 | Yes | Yes |
| Planctomycetes Planctomycetia | Yes | Yes |
| Proteobacteria Alphaproteobacteria | Yes | Yes |
| Proteobacteria Betaproteobacteria | Yes | Yes |
| Proteobacteria Deltaproteobacteria | Yes | Yes |
| Proteobacteria Gammaproteobacteria | Yes | Yes |
| TM6 SJA-4 | Yes | Yes |
| Verrucomicrobia Pedosphaerae | Yes | Yes |
| Verrucomicrobia Opitutae | Yes | Yes |
| Verrucomicrobia Verrucomicrobiae | Yes | Yes |
